# Hirsutellin A: A Paradigmatic Example of the Insecticidal Function of Fungal Ribotoxins

**DOI:** 10.3390/insects4030339

**Published:** 2013-07-09

**Authors:** Elías Herrero-Galán, Lucía García-Ortega, Miriam Olombrada, Javier Lacadena, Álvaro Martínez del Pozo, José G. Gavilanes, Mercedes Oñaderra

**Affiliations:** Departamento de Bioquímica y Biología Molecular I, Universidad Complutense, 28040 Madrid, Spain; E-Mails: eherrero@cnb.csic.es (E.H.-G.); lucia@bbm1.ucm.es (L.G.-O.); miriam@bbm1.ucm.es (M.O.); jlacaden@quim.ucm.es (J.L.); alvaromp@quim.ucm.es (A.M.P.); ppgf@bbm1.ucm.es (J.G.G.)

**Keywords:** insecticidal proteins, ribotoxins, α-sarcin, hirsutellin, ribonucleases, cytotoxic proteins

## Abstract

The fungal pathogen *Hirsutella thompsonii* produces an insecticidal protein named hirsutellin A (HtA), which has been described to be toxic to several species of mites, insect larvae, and cells. On the other hand, on the basis of an extensive biochemical and structural characterization, HtA has been considered to be a member of the ribotoxins family. Ribotoxins are fungal extracellular ribonucleases, which inactivate ribosomes by specifically cleaving a single phosphodiester bond located at the large rRNA. Although ribotoxins were brought to light in the 1960s as antitumor agents, their biological function has remained elusive. Thus, the consideration of hirsutellin A, an insecticidal protein, as a singular ribotoxin recalled the idea of the biological activity of these toxins as insecticidal agents. Further studies have demonstrated that the most representative member of the ribotoxin family, α-sarcin, also shows strong toxic action against insect cells. The determination of high resolution structures, the characterization of a large number of mutants, and the toxicity assays against different cell lines have been the tools used for the study of the mechanism of action of ribotoxins at the molecular level. The aim of this review is to serve as a compilation of the facts that allow identification of HtA as a paradigmatic example of the insecticidal function of fungal ribotoxins.

## 1. Introduction

Toxins are molecules affecting vital physiological systems. Many toxins have been explored and developed into drugs for the treatment of many different diseases and others are still under study with the same purpose. But one question to answer is why do organisms need to produce toxins?

All living beings develop several strategies to survive, including a variety of mechanisms for defense, preying, and feeding. Fungi have traditionally been a major source of toxins for study and biotechnological application. They are a rich source of nitrogen and phosphorous and suffer constant attacks by fungivorous animals such as mites, insects, and arthropods [1,2,3]. Thus, fungi secrete a wide variety of toxins with quite different purposes [4]. Fungal ribotoxins were discovered in the 1960s during a screening program searching for antibiotics and antitumor agents. A protein inhibitory to sarcoma 180 and carcinoma 755 induced in mice was found in the culture filtrates of the mold *Aspergillus giganteus*, isolated from a sample of farm soil. The protein responsible for these effects was named α-sarcin after its anti-sarcoma activity [5]. A few years later it was demonstrated that their mechanism of action was based on their ability to inhibit protein biosynthesis [6,7]. This fact prompted the molecular characterization of ribotoxins as a widespread group of highly specific ribonucleases produced by filamentous fungi, which induce cell death by ribosome inactivation. They are extracellular proteins, which behave as potent inhibitors of protein biosynthesis in almost any organism [8,9,10]. They cleave one single and unique phosphodiester bond located within the universally conserved sarcin-ricin loop (SRL) of the large rRNA [6,11,12], leading to cell death by apoptosis [13].

Nowadays, four of these ribotoxins have been thoroughly studied and characterized: α-sarcin, the first one to be discovered [5,9]; restrictocin, with similar activities to those of α-sarcin [9,10]; AspF1, a major allergen in *Aspergillus* related diseases [14,15]; and more recently, hirsutellin A, the smallest ribotoxin known. Although ribotoxins were initially discovered as antitumor molecules, further studies revealed an unspecific cytotoxicity of these proteins, which limited their potential clinical uses [16] and caused the abandonment of their study as toxic molecules.

The biological function of ribotoxins has been a matter of speculation since their discovery. Apart from the generalized idea of being involved in defense or predation, the assignment of an insecticidal function for fungal ribotoxins has been pointed out [17,18]. In this sense, it has been suggested that they could protect the ribotoxin-producing fungi by deterring insect feeding on their phialides [17]. Accordingly, it was proposed that ribotoxin production would be related to fungal protection against arthropods, mainly mites and insects. For example, beetles of the *Carpophilus freemani* species that are able to feed on *Aspergillus restrictus,* the restrictocin-producing organism, could not feed upon this fungus during conidia maturation. On the other hand, beetles were not able to feed on *Aspergillus nidulans* genetically modified to produce the ribotoxin by transforming the fungus with the cDNA of restrictocin placed under the control of the glucoamylase promoter [18]. This protection was not observed in the wild-type fungus in which the genome does not contain any ribotoxin gene [17]. Accordingly, it was also observed that ribotoxins were accumulated on the conidia surface upon maturation of *A. restrictus* [19,20].

In relation to ribotoxin biosynthesis, little is known about the mechanisms by which the producing fungi might protect themselves from their own toxicity. Lamy and Davies [21] suggested that prorestrictocin might be inactive until the protein is processed during secretion, but Yang and Kenealy [22] showed that neither the leader sequence nor the putative prosequence inhibited the action of the cytotoxin under *in vitro* or *in vivo* conditions. These authors suggest that restrictocin could be sequestered in membrane systems and transported to certain locations or secreted outside. Immunofluorescence studies on the localization of restrictocin in *A. restrictus* support this idea. An alternative hypothesis to the active secretion is that an inactivating protein could bind restrictocin until its liberation, but no evidence exists to support such a system in *A. restrictus*.

The fungal genus *Hirsutella* has over 50 species that are able to colonize a wide variety of insects. Several studies have reported that crude filtrates of a particular species of this genus denominated *Hirsutella thompsonii* were toxic to various groups of arthropods as moth, fly and mosquito larvae, aphids, and mites [23,24], causing their death. This fungus is a specific fungal pathogen of *Acarina* inhabiting citrus and other plants in most subtropical and tropical regions [25] and had been previously developed as a microbial insecticide for use against the mite *Phyllocoptruta oleivora* [26]. The insecticidal action of this invertebrate fungal pathogen has been extensively documented [27,28] 

The insecticidal protein HtA was originally detected in 1995 while looking for the effective toxic agents produced by *Hirsutella thompsonii* [28,29]. It has been reported that HtA preparations are highly toxic *in vivo* to the adult citrus rust mite, *Phyllocoptruta oleivora*, the natural host to this parasitic fungus [26]. Moreover, HtA is lethal to *Galleria mellonella* larvae [29] and produces cytopathic effects on certain insect cell lines such as *Spodoptera frugiperda* cells [27]. HtA also inhibits the protein synthesisof the Brome mosaic virus in both rabbit reticulocyte and wheat germ *in vitro* translation system [27].

Recently, evidence has been found that HtA is able to reproduce the specific ribonucleolytic action and other abilities of ribotoxins, and so it has been included as a new member of the α-sarcin/restrictocin family. Although the question of whether HtA is just an exception within the ribotoxin family has been proposed, both its insecticidal and its ribotoxin activities have been clearly demonstrated [26,28,29,30,31]. The discovery of this smaller HtA ribotoxin has revived the old proposal that insecticidal ability could be the long searched for natural function of the fungal ribotoxins family. The studies performed comparing the activities of HtA and α-sarcin against insect larvae and cells [31] have shown that HtA could be the demonstration that invertebrate pathogenic activity is the biological function of the ribotoxins family. In this regard, the study of HtA represents an important milestone in the knowledge of the structure, function, and diversity of fungal ribonucleases.

## 2. Ribotoxins Evolution: Role of HtA

Ribotoxins are an intriguing group of proteins regarding their evolution and structure-function relationships [32,33]. They belong to the barnase superfamily, formed by small ribonucleases consisting of only one polypeptide chain [34]. This superfamily also includes non-toxic unspecific RNases of the T1 family. The high degree of sequence and structural similarity between ribotoxins and T1-like RNases has led to the suggestion that both families could have a common ancestor [32,33]. This similarity includes the active site responsible for the phosphodiesterase activity of these enzymes [35]. RNase U2, produced by the fungus *Ustilago sphaerogena*, stands out as the unspecific fungal extracellular RNase most closely related to ribotoxins [36,37]. It displays 34% sequence identity with the α-sarcin family, and it is 10 residues longer than other T1-like RNases (114 *vs.* 101). Ribotoxins also share, with RNases of the T1 family, their main structural core, but they present a number of characteristics that make them unique within the whole superfamily. Ribotoxins are basic proteins, and they are around 40 residues longer (140–150 amino acids). The main structural differences between ribotoxins and RNases of the T1 family are the length and arrangement of the non-ordered protein loops and the N-terminal β-hairpin, which are positively charged in ribotoxins. Thus, these regions are supposed to be the determinants of the extra activities of ribotoxins, such as their specificity and cytotoxicity [38]. The ability to enter cells and to display specific ribonucleolytic action against a single phosphodiester bond in the whole ribosome distinguishes ribotoxins from non-cytotoxic relatives of the T1 family. That is to say, ribotoxins are extremely specific ribonucleases when compared to the non-toxic counterparts of the T1 family of fungal extracelular ribonucleases.

**Figure 1:**
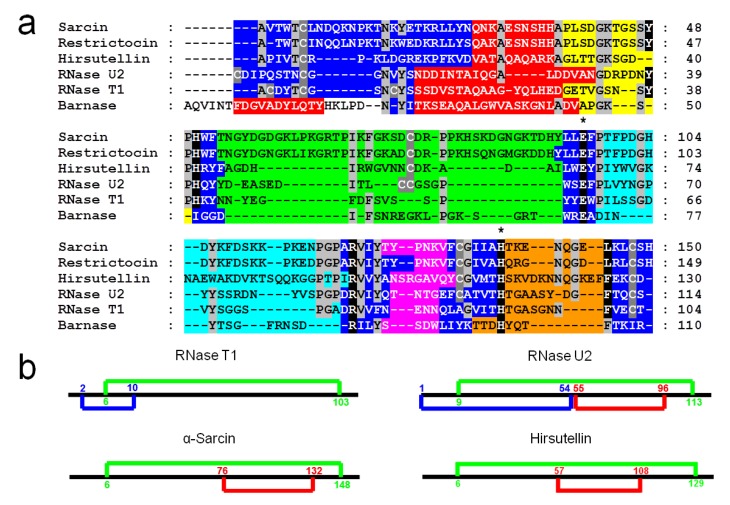
(**a**) Alignment of the amino acid sequences of α-sarcin, restrictocin, hirsutellin A, RNase U2, RNase T1, and Barnase. Conserved residues (light grey boxes) in at least four sequences are enlightened, as well as the cysteine residues (dark grey boxes). Residues implicated in the active site are highlighted in black and essential catalytic residues are remarked by (*). Elements of secondary structure are displayed by colors: β-hairpin, (dark blue boxes), residues at the helical portion, (red boxes) and residues in loops 1, 2, 3, 4, and 5 (yellow, green, light blue, pink, and orange boxes, respectively). (**b**) Comparison of the disulfide bridges arrangement in the Barnase superfamily.

**Figure 2:**
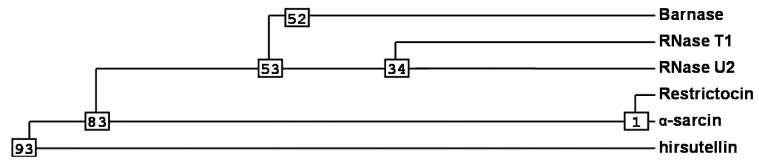
Phylogenetic analysis [42] for the most important members of the Barnase superfamily. Numbers shown in the phylogram are distances corresponding to the amino acid sequence alignment of Figure 1.

Ribotoxins are considered to be naturally engineered proteins that evolved from nontoxic ribonucleases [10]. They exhibit a high degree of identity (above 60%), including two disulphide bridges conserved along the whole family (Figure 1) [9,37,39,40]. Interestingly, HtA shares this characteristic, although it is 20 residues shorter than the other ribotoxins and shows only 25% sequence identity with previously known members of the family [37]. These were the reasons why HtA appeared initially after its discovery as a feasible candidate to be an evolutionary intermediate between T1-like RNases and ribotoxins (Figure 2). However, the further characterization of HtA showed that it maintains all the ribotoxin abilities, proving that these can be accommodated into a shorter amino acid sequence [30,41]. Thus, it has been suggested that HtA could actually be a refined ribotoxin that would have evolved further in order to become smaller and more economical.

## 3. Structural Features

Ribotoxins and non-cytotoxic ribonucleases of the RNase T1 family show high structural homology but different specific activities. Although classic ribotoxins, such as α-sarcin and restrictocin (about 150 amino acids), are larger than non-toxic RNases (about 96–110 amino acids), they share a similar central structure region, including a conserved β-sheet core composed of five strands connected by loops of different lengths. Structural differences between both families are mainly concentrated at these loops and the N-terminal β-hairpin. Consequently, they have been proposed to be the structural determinants responsible for ribotoxin toxic properties [35]. The three-dimensional structures of restrictocin [43] and α-sarcin are known [35,44]. Furthermore, the characterization of a high number of α-sarcin mutants, including bidimensional nuclear magnetic resonance (NMR) studies [45,46], have been the means used to make a very detailed map of its structural and functional properties.

After its discovery as the molecule responsible for the insecticidal activity of the entomopathogenic fungus *Hirsutella thompsonii*, the cDNA of HtA was cloned and sequenced [47]. The corresponding amino acid sequence alignment with other microbial RNases and ribotoxins suggested that the common structural core was also conserved in HtA, the most significant differences being again the length of the loops connecting the α-helical and the β-sheet regions [37]. These loops in HtA were predicted to be longer than the corresponding ones in small microbial RNases, but shorter than those in ribotoxins. In addition, the four cysteine residues involved in two disulfide bridges [35,37] as is the case for α-sarcin, were conserved in HtA (Figure 1b).

**Figure 3:**
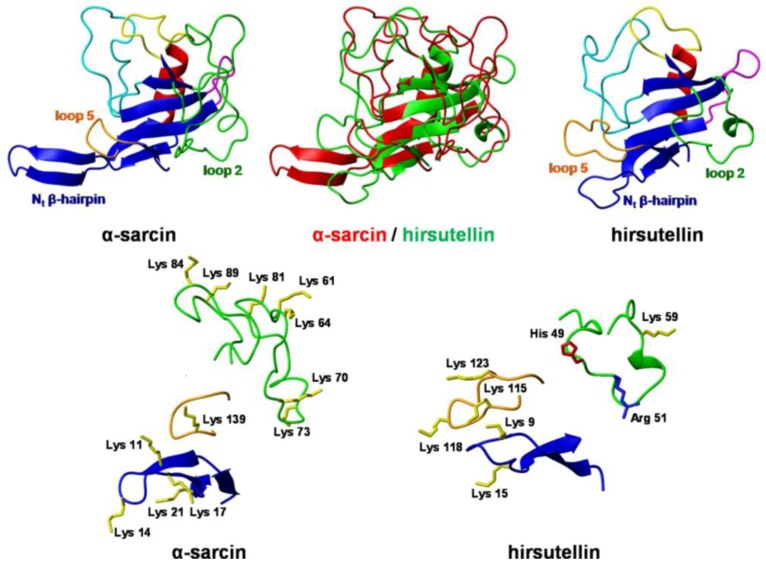
Three-dimensional structures of the ribotoxins α-sarcin and hirsutellin A (HtA) Spatial orientation of the N-terminal β-hairpin and loops 2 and 5 in HtA and α-sarcin. Positively charged residues are depicted. Color code is the same as in Figure 1. The diagrams were generated using MOLMOL [49].

From the clone of the cDNA of HtA, plasmid pTac-TacHtA was constructed for the expression of mature HtA in *E. coli* [30]. HtA was purified both from its natural source and also as a recombinant protein. Spectroscopic analysis determined that the fungal protein and the recombinant one were indistinguishable. The exhaustive characterization of both forms of HtA reveals an E coefficient (0.1%, 280 nm, 1 cm) of 2.00 for both proteins [30]. The mid-point of the thermal denaturation transition (T_m_) determined by circular dichroism (CD), and differential scanning calorimetry (DSC) was 62 ºC. This value was 10 ºC higher than that reported for α-sarcin [30] but closer to 61 ºC and 59 ºC, the T_m_ values for the ribotoxins AspF1 and restrictocin, respectively [48]. In 2009, the elucidation of the three-dimensional structure of HtA in solution by nuclear magnetic resonance (NMR) [41] confirmed that the overall protein fold of ribotoxins is maintained in this smaller polypeptide chain, but some important differences apart from size-derived variations were observed. The structure consists of one α-helix, one helical turn, and seven β-strands that form a β-sheet and a N-terminal hairpin, with a characteristic α + β fold and a highly positively charged surface. The most relevant structural differences when compared to its larger homolog, α-sarcin, are the shorter lengths of loop 2 and the N-terminal β-hairpin, which is also less positively charged (Figure 3). This truncation and reduced charge of the N-terminal hairpin in HtA may be compensated by the extension and different orientation of its loop 5, which exhibits a higher amount of positively charged residues.

**Figure 4:**
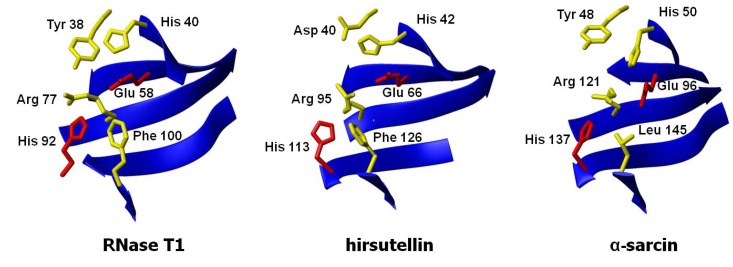
Three-dimensional structures of the active sites of ribotoxins α-sarcin and hirsutellin A (HtA) and the non-toxic fungal RNase T1. The structures were fitted to the peptide bond atoms of the active site residues of α-sarcin (His 50, Glu 96, and His 137) and RNase T1 (His 40, Glu 58, and His 92) and those at homologous positions in HtA (His 42, Glu 66, and His 113).

As far as the active site is concerned, that of α-sarcin is well known (Figure 4). Among these residues, His 50, Glu 96, and His 137 form the catalytic triad. The equivalent residues in HtA (His 42, Glu 66, and His 113) have been identified by comparison of its three-dimensional structure with that of other ribotoxins (Figure 4). The essential residues for ribonuclease activity are conserved, but some other features are closer to T1-like RNases (like the presence of a Phe residue instead of α-sarcin’s Leu 145) or even completely new in the whole superfamily (an aspartate group at a position equivalent to α-sarcin’s Tyr 48, for example) [41]. Several substitution mutants of this region were studied regarding their implication in the functionality of the protein, in order to shed new light on the requirements for ribotoxin activity [50]. Within this idea, a region was found to exhibit significant differences with α-sarcin, related to Trp 71 and Trp 78 in HtA. Studies with single and combined mutants of these two residues revealed that this region seems to be involved in the higher membrane permeabilizing activity of HtA when compared with the other members of the ribotoxins family. The W71/78F mutation in HtA resulted in a loss of cytotoxicity, but maintained the ribonucleolytic specific activity [51]. These residues are not conserved in α-sarcin. It has been postulated that a β-structure region comprising residues 116–139 could be involved in the hydrophobic interaction of α-sarcin with membranes [52].

## 4. Functional Properties

It is well known that ribotoxins are cyclizing ribonucleases that exhibit a two-step enzymatic mechanism [9,30,53]. First, they form a 2’,3’-cyclic phosphate intermediate via a transphosphorylation reaction and then hydrolyze this intermediate to the corresponding 3’-phosphate [53].

Their toxicity arises from their ability to cross cell membranes and, once inside the cell, impair ribosome function by cleaving a single phosphodiester bond, that between G4325 and A4326 in 28S rRNA (rat ribosome numbering) at the sarcin/ricin loop (SRL) [9]. This cleavage interferes with the function of elongation factors, producing protein biosynthesis inhibition and cell death by apoptosis [13,54]. This highly specific ribonucleolytic activity of ribotoxins has been extensively studied and a wide collection of mutants has been well characterized. Thus, the reaction mechanism, as well as the roles of most of the active site residues, have been elucidated [55,56,57,58,59,60].

HtA produces the same cleavage as α-sarcin but shows two-fold higher specific activity when assayed against eukaryotic ribosomes. HtA is also able to cleave 35mer synthetic SRL-RNA, liberating 14mer and 21mer oligonucleotides, with identical activity values than those of α-sarcin [30]. Residues forming the catalytic triad of HtA (His 42, Glu 66, and His 137), as well as the outstanding Asp 40 (Figure 4), have been studied in order to shed new light on the requirements for ribotoxin activity. Seven substitution mutants, H42Q, E66Q, and H113Q, as well as double and triple mutants in all possible combinations, were produced and characterized regarding their ribonucleolytic activity and cytotoxicity [50]. Implication of these three residues in the ribotoxin activity of HtA was confirmed, though none of them resulted strictly essential for ribosomal cleavage. An Asp residue (Asp 40 of HtA) is now highlighted as a novelty in this field. It has been clearly established that the catalytic process in ribotoxins is extremely dependent on a precise structural and electrostatic environment of the active site [55,58,59,61]. Studies with mutants D40N and D40N/E66Q demonstrated an important role for Asp 40 in the activity of the protein and revealed a new set of electrostatic interactions quite different from the one described for already known ribotoxins [50], giving to this Asp residue a unique role among the other members of the family. 

Although passage through the cell membrane is the first and limiting step for ribotoxin cytotoxicity [62], knowledge about the mechanism of cell entry is scarce. The most relevant data concerning this topic have been obtained for α-sarcin and HtA. This ability to enter cells is also the main functional difference between ribotoxins and their non-cytotoxic relatives microbial RNases from the T1 family. After internalization by endocytosis, ribotoxins reach the cytosol by clathrin-independent transport via acid endosomes and the Golgi [13]. The positively charged surface of ribotoxins seems to be a key factor for this cytotoxicity through interaction with the lipid membrane components since no protein receptor has been found [63,64,65,66]. Studies with vesicle-model systems have shown that α-sarcin specifically interacts with acid phospholipid vesicles of phosphatidylserine or phosphatidilglycerol at neutral pH [8,66,67,68] and promote their aggregation, with the protein acting as a bridge between lipid bilayers [69,70,71]. This fact would be in agreement with the preference exhibited by ribotoxins for virus-infected or tumor cells, with a higher exposure of acid phospholipids to the extracellular medium due to a loss of symmetry in the plasma membrane [72,73,74,75,76] or a higher content of negatively charged phospholipids, such as phosphatidylserine [77,78]. The involvement in malignant transformation of the enzymes responsible for phosphatidic acid synthesis (diacylglycerol kinases) seems to further support this hypothesis [79,80,81]. All previous observations are consistent with a membrane interacting mechanism involving an intercalation of the ribotoxins into the lipid matrix, which would promote fusion and permeability changes in the bilayers, processes that would presumably be involved in the ribotoxin passage across the membranes of its target cells [8]. 

The ability to interact with lipid membranes has been associated with the non-ordered protein loops and the N-terminal β-hairpin of ribotoxins (Figure 3), where the main structural differences between ribotoxins and non-cytotoxic RNases of the T1 family are located. In this regard, deletion of α-sarcin’s N-terminal β-hairpin produces an active ribonuclease with altered membrane interaction properties [82,83]. Close to this region, the structure of HtA presents two Trp residues, the above-mentioned Trp 71 and Trp 78, which seem to be taking active part in its higher membrane permeabilizing activity. Studies with single and combined mutants of these two residues provide evidence that cell membrane passage and internalization, as well as substrate specific recognition, require the participation of this region. Additionally, mutant W71/78F has been the first non-cytotoxic but specific ribosome-cleaving ribotoxin obtained to date [51], which has revived the old interest for the potential biomedical application of ribotoxins. One of the classic goals of the study of ribotoxins has been the construction of immunoconjugates with a tumor-specific targeting moiety [84,85], but the non-specific toxicity exhibited by ribotoxins against non-tumoral cells has always been a major concern for researchers in this field. Therefore, HtA W71/78F, which retains the specific ribonucleolytic activity of ribotoxins without their unspecific ability to enter cells, appears as a feasible candidate for the construction of specific and safer immunotoxins, as their *in vivo* cytotoxicity would only be manifested against cells targeted by the conjugated antibody.

## 5. Insecticidal Activity

The assignment of an insecticidal function to fungal ribotoxins has been suggested before [17,18]. Several studies have demonstrated that *Hirsutella thompsonii* is able to infect arthropods [23,24]. Under *in vivo* conditions, conidia contact the host, attach to the cuticle, germinate, and penetrate through it [27]. The potential of *Hirsutella thompsonii* as a biological control agent of the parasitic mite *Varroa destructor*, a honey bee parasite, has also been shown [86,87].

Insect cells have a different plasma membrane composition from mammalian cells, with a higher content of phosphatidylethanolamine and phosphatidylinositol and a significant lower cholesterol/phospholipid ratio [88]. Thus, insect plasma membranes show different permeability. Probably these membranes are thinner and more fluid than those in mammalian cells, being better candidates as ribotoxin targets.

**Figure 5:**
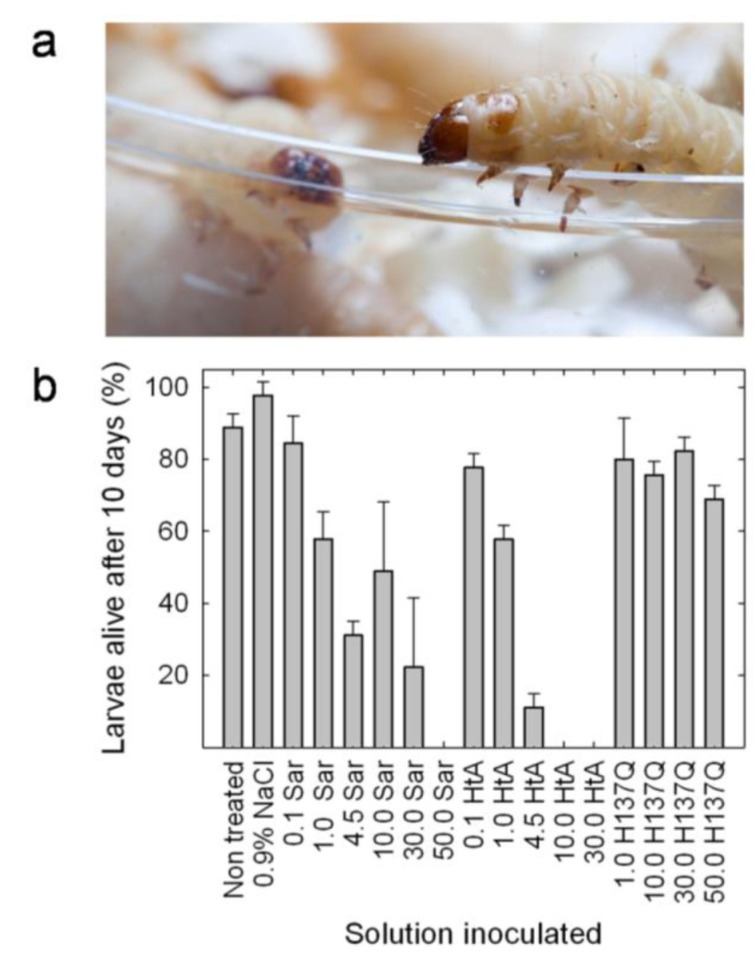
**(a)** *Galleria mellonella* larvae; **(b)** Histogram representing the survival of *G. mellonella* larvae after ten days of being injected with 8 μL of 0.9% NaCl containing different (μM) concentrations of wild-type α-sarcin, a catalytically inactive mutant (H137Q) or wild-type HtA. The initial number of individuals in each group represented was 15. Results correspond to the average of three independent experiments. Bars represent the standard deviation error.

The insecticidal activity of hirsutellin has been well documented [29]. However, no proven data of this activity have been obtained for any of the other members of the ribotoxin family although some studies had suggested that insecticidal activity could be the natural function of ribotoxins [17,18]. Therefore, a study comparing HtA and α-sarcin has been recently performed with the goal of elucidating this idea [31]. The results obtained show that both α-sarcin and hirsutellin A are highly toxic against *G. mellonella* larvae (Figure 5). Injection of α-sarcin or HtA caused larvae death and pupation delay. Virulence was dependent on ribotoxin concentration. Data analysis revealed that there was statistically significant difference between the treatment with α-sarcin and HtA, being HtA more effective in terms of less amount of protein needed to produce the same death levels. Indeed, injection of the catalytically inactive α-sarcin H137Q mutant [56] had an almost negligible effect on survival for identical incubation times and doses, correlating toxicity with the ribonucleolytic activity of these proteins.

This result suggests that the insecticidal lethal action of ribotoxins is dependent on their highly specific RNase activity. Injection of the toxin produced loss of larvae mobility followed by larvae death, being the differences in larvae survival more evident after 10 days of incubation at 30 ºC [31]. Larvae coloration changed from brownish to dark brown or even black upon death (see complementary video material). These color changes were most probably a consequence of the overactivation of the phenoloxidase cascade, a process that has been proposed as one of the most important defense mechanisms against pathogens in insects [89]. The toxic effect of both ribotoxins was also evident in the observed pupation delay. 

**Figure 6:**
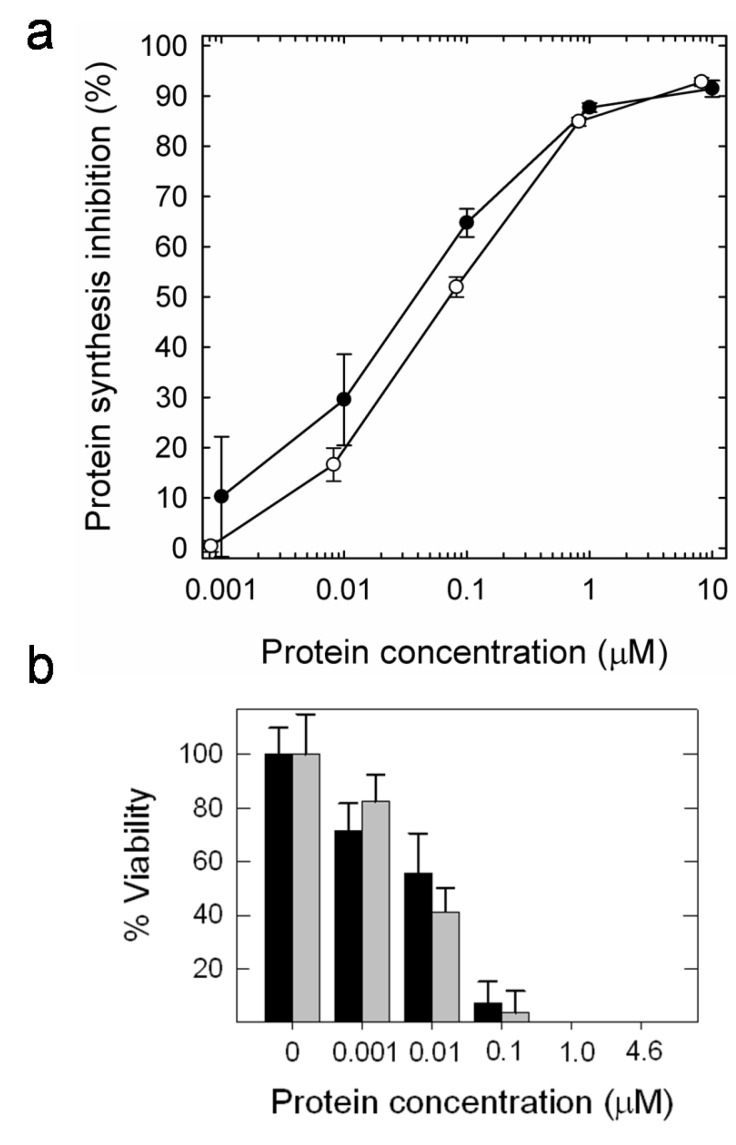
(**a**) Protein biosynthesis inhibition in Sf9 insect cells cultured in the presence of increasing HtA (open circles) or α-sarcin (dark circles) concentrations. Semilogarithmic plots are the average of three different sets of experiments. Bars represent the standard deviation. **(b)** Toxic effect in Sf9 insect cells after the addition of different HtA (grey bars) or α-sarcin concentrations (black bars), expressed in terms of percentage of cell viability after 60 hours of incubation with the protein.

Both ribotoxins are also highly toxic against insect cells in culture (Figure 6). Two different insect cell lines have been assayed, *Spodoptera frugiperda* (Sf9) and *Trichoplusia ni* (High Five). Both α-sarcin and HtA cause a dramatic effect on both inhibition of *in vivo* protein biosynthesis and decreasing cell viability (Table 1). It is remarkable that the IC_50_ values were almost two orders of magnitude smaller than the corresponding values obtained for these ribotoxins against human rhabdomyosarcoma cells [13,30,62], the tumor cell line commonly used as the reference assay for evaluating the antitumoral activity of ribotoxins. 

**Table 1:** IC_50_ values obtained for α-sarcin and HtA when assayed against insect cell lines in culture.

	Viability		Protein biosynthesis inhibition
	Sf9	High Five	Sf9
**α-sarcin**	0.010 µM	0.100 µM	0.04 µM
**HtA**	0.010 µM	0.025 µM	0.07 µM

Moreover, the insect killing activity of both ribotoxins is related to their specific ribonucleolytic action on insect ribosomes. The characteristic activity of ribotoxins is manifested by the production of a 400 nt fragment (α-fragment) upon degradation of the target ribosomes. In fact, the production of α-fragment was observed in treated insect cells. The results revealed the cleavage of 28S rRNA by both ribotoxins with an identical pattern as that described for isolated ribosomes [54]. These data support the idea that the specific ribonucleolytic activity of HtA and α-sarcin is the reason of their toxicity against this insect cell lines. Corroborating that specific ribotoxin action involves the passage of the protein across the membranes of its target cells, both toxins also retained the membrane interaction ability when tested against model vesicles made of insect plasma membrane lipids [31].

Finally, the long studied antitumoral activity of ribotoxins could be a side effect of their insecticidal action and ribotoxins could be considered host-defense proteins and thus included in the new generation of bioinsecticides [90,91]. 

## 6. Conclusion

Hirsutellin A (HtA) has been recently demonstrated to be the smallest ribotoxin known to date [30]. It is 20 residues shorter than previously described ribotoxins (130 amino acids compared to 149/150) and exhibits only 25 percent sequence identity to other members of this family. However, it exhibits all the ribotoxin abilities. HtA is able to specifically degrade ribosomes releasing the 400nt α-fragment typically detected after ribotoxin activity. As its larger relatives, it also produces this single cleavage on short synthetic oligonucleotides mimicking the sequence and structure of the SRL [9]. Like these ribotoxins, HtA is highly basic, possesses cytotoxic activity and inhibits protein synthesis [28]. In conclusion, HtA seems to be a more evolved ribotoxin displaying all the functions of ribotoxins in a shorter polypeptide chain [30].

In addition, HtA displays a well-characterized insecticidal biological action [27]. Interestingly, several studies relating ribotoxin expression and conidiophore maturation suggest that ribotoxins may play a defensive role against arthropods like insects or mites [17]. Thus, the characterization of HtA as a ribotoxin may support the theory that they are involved in defense mechanisms for either the producing mold or the plants where they live in symbiosis. We have unequivocally confirmed the insecticidal properties of ribotoxins [31]. First, the interaction of α-sarcin and HtA with insect ribosomes, isolated from *Spodoptera frugiperda* Sf9 cell cultures showed specific ribonucleolytic activity. Second, their insecticidal activity against two different insect cell lines (*Spodoptera frugiperda* Sf9 and *Trichoplusia ni* High five) has been proved and directly related to the catalytic production of the α-fragment. Finally, both ribotoxins exhibit high toxicity when injected at low doses into *G. mellonella* larvae. According to this, it seems reasonable to think that ribotoxins evolved from non-specific RNases driven by the necessity of fungal defense against insects. In this regard, it is very interesting to consider the fact that insect cell membranes show many of the characteristics of transformed cells and, thus, the insecticidal biological role of ribotoxins could also explain their intriguing antitumoral properties.

In relation to the production of potential insecticidal agents based on ribotoxins, further work must be carried out. Pure restrictocin (1000 p.p.m.) added to the diet of *Carpophilus freemani*, a fungus-feeding beetle, killed 38.5% of larvae in 48 h. Adult *C. freemani* were not affected, but they exhibited an aversion to restrictocin in their diet [17]. In this sense, HtA and α-sarcin could be used as insecticidal agents, but new studies must be established in order to assess a suitable way of toxin administration based on feeding habits. Ribotoxin concentration and digestion control of the proteins ingested should be considered. 

Although the unspecific cytotoxic action of ribotoxins could limit their potential use as insecticidal agents, the present accumulation of data about their mechanism of action allow an optimistic view. In this regard, the development of immunotoxins based on these fungal ribonucleases stands out as an alternative in the mid-term future. Nowadays, second generation immunotoxins, based on the fusion of ribtoxins to a single chain containing only the variable domains needed for antigen recognition, have been already obtained [92]. In this sense, a colon cancer-specific immunotoxin, based in α-sarcin, has been recently produced and characterized [84]. These studies also open a new way to solve the potential disadvantages of the application of ribotoxins as insecticides, as conjugation of the toxin to an insect-specific antibody would avoid damage to the beneficiary species. On the other hand, the design of adequate mutants and chimaeric ribotoxins with convenient activities must also be considered.

## Supplementary Files

Supplementary File 1
